# Three-dimensional evaluation of the spatial morphology of stented coronary artery segments in relation to restenosis

**DOI:** 10.1007/s10554-019-01628-3

**Published:** 2019-05-24

**Authors:** Áron Üveges, Csaba Jenei, Tibor Kiss, Zoltán Szegedi, Balázs Tar, Gábor Tamás Szabó, Dániel Czuriga, Zsolt Kőszegi

**Affiliations:** 1 Division of Cardiology, Department of Cardiology, Faculty of Medicine, University of Debrecen, Debrecen, Hungary; 2 Szabolcs – Szatmár – Bereg County Hospitals and University Teaching Hospital, Nyíregyháza, Hungary

**Keywords:** Arc-chord ratio, In-stent restenosis, 3D reconstruction, Bending angles

## Abstract

To investigate the correlations between the three-dimensional (3D) parameters of target coronary artery segments and restenosis after stent implantation. Sixty-four patients after single, cobalt chromium platform stent (27 BM stents and 37 DES) implantation were investigated retrospectively 12 ± 6 months after the index procedure. 3D coronary artery reconstruction was performed before and after the stent implantation using appropriate projections by a dedicated reconstruction software. The curve of the target segment was characterized by the ratio of the vessel length measured at midline (arc: A) and the distance between the edge points of the stent (chord: C): A/C ratio (ACr). Age, diabetes and hyperlipidaemia were taken into account for the statistical evaluation. 22 patients were diagnosed with ISR, while 42 patients without any restenosis served as controls. The two groups did not differ regarding major cardiovascular risk factors, proportion of the treated vessels or the type of stents. Higher initial ACr values were associated with greater straightening of the vessel curvature in all groups (p < 0.001). Significant negative correlations were found in cases of proximal or distal edge bending angles (p < 0.001). Pre-stent edge bending angles < 7° often showed an increase after the stent implantation, while in case of higher initial values, the bending angles generally decreased. Using multivariate logistic regression modelling we found that the pre-stent ACr was an independent predictor of in-stent restenosis (odds ratio for 1% increase of the ACr: 1.08; p = 0.012). Changes of angles at the stent edges following stent implantation correlate with the initial local bending angles. The ACr predispose to chronic shear stress in the vessel wall, which may contribute to the pathological intimal proliferation.

## Introduction

Over the last three decades, stent implantation became the most widely performed procedure for the treatment of symptomatic coronary artery disease [1]. Despite the new drug eluting devices, in-stent restenosis (ISR) remained the leading cause of late stent failure. The incidence of ISR depends on the clinical characteristics of the patient and may reach 5–10%. The risk factors can be divided into systemic (e.g. diabetes mellitus), procedural (e.g. underexpansion of the stent) and local vessel determinants. Regarding local features, the design, the length and the diameter of the stent were shown to be independent predictors of ISR. The vascular tortuosity was also proven to be an important factor [2].

Lesions located in severely angulated coronary artery segments have been associated with an increased risk for major adverse cardiac events after stent implantation [3, 4]. The mechanism behind this phenomenon was proposed to be the change of the wall shear stress, as a contributor of intimal hyperplasia [5–7]. However, the question “whether the detected intimal hyperplasia is a part of the healing process after stent implantation or a predictor of later clinical restenosis” has not yet been answered [8].

Conventional two-dimensional (2D) coronary angiography may limit the characterisation of the actual impact of coronary vessel tortuosity. Instead, three-dimensional (3D) reconstruction is required to adequately recognise the anatomy and spatial run of the curved segment in question. Despite the specific software packages on the market with 3D reconstruction algorithm allowing an accurate geometric analysis of the tortuous coronary artery [9, 10], the literature contains limited data about the impact of 3D coronary artery geometry on ISR, and conclusive data are lacking regarding the detailed effect of stent implantation on the 3D geometric changes.

The purpose of this study was to explore potential links between the data of the 3D analysis and late stent failure.

## Methods

### Study design

In this retrospective, multicentre study, we screened patients referred for a repeat coronary angiography 3–30 months following stent implantation. Cardiac catheterisations were performed in the haemodynamic laboratory of the Department of Cardiology and Cardiac Surgery, University of Debrecen and Jósa András Teaching Hospital, Nyíregyháza between 1st January and 31st December 2015. All data were retrospectively analysed from the hospital information system and from the local PACS database. All patients signed a written informed consent and the study protocol was approved by the local ethics committees.

The main inclusion criteria were the presence of an implanted stent with a length ≥ 18 mm and the availability of at least two different angiographic images recorded from the target coronary artery segment ≥ 25° apart. Patients either with bare-metal (BMS) or drug-eluting stents (DES) were included. The stent platform was restricted to that of cobalt-chromium, which assured a quite homogeneous group regarding the mechanical characteristics of the stent. Patients, in whom the stent was implanted in a coronary artery bypass graft were excluded, as well as cases with poor image quality or unsuitable images for 3D reconstruction (Table 1). Altogether 64 patients fulfilled all criteria (mean age: 65 ± 9 years).

**Table 1:** Inclusion and exclusion criteria for patient selection

Inclusion criteria
Angiographic evidence of in-stent restenosis ( > 50% diameter stenosis)
Stent length ≥ 18 mm
2 recordings of the examined coronary artery segment from different angiographic projections ≥ 25° apart
Stent type: bare-metal stent (Multilink Vision, PRO-Kinetic Energy, Integrity), drug-eluting stent (Xience-V, Orsiro, resolute integrity)
Exclusion criteria
Stent implanted in a coronary artery bypass graft
Unsuitable images (e.g. foreshortening of the target coronary artery segment, overlapping of other segments)
Bioresorbable vascular scaffold (BVS)
Poor image quality (unable to follow luminal border)

### 3D reconstruction

**Fig. 1:**
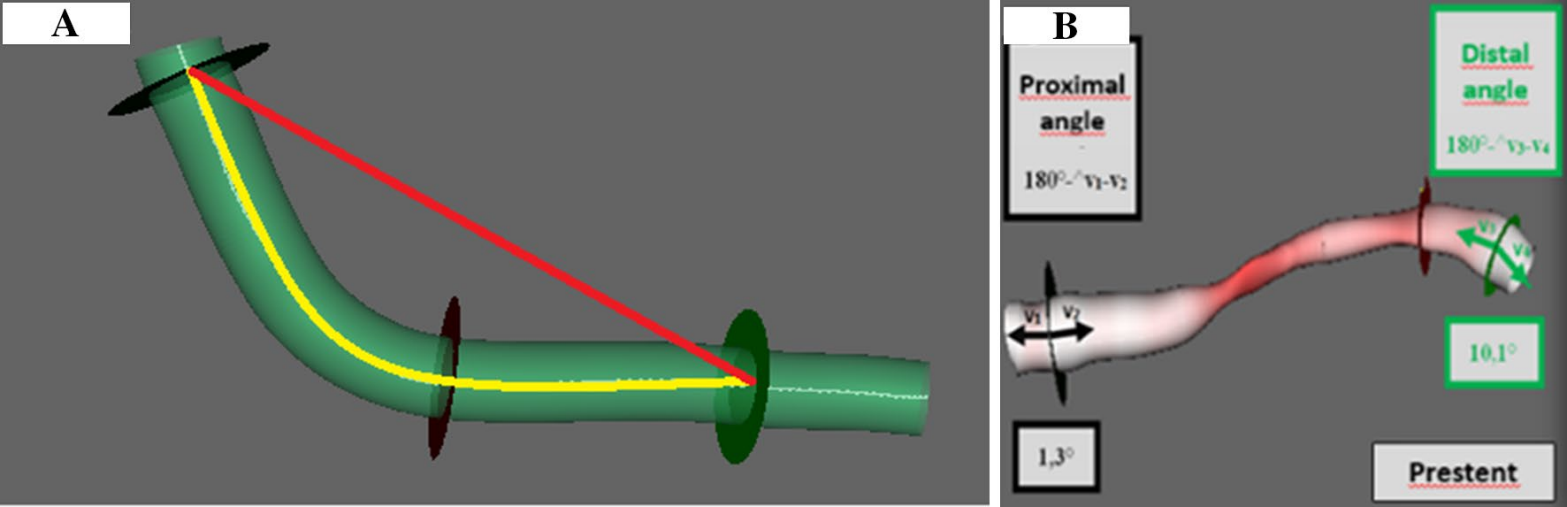
Calculation of the arc-chord ratio (**a**) and edge bending angles (**b**). The arc is represented by the midline of the analysed segment (yellow), the chord is represented by the straight red line (**a**). To calculate the bending angles two vectors were created along the centreline of the vessel towards the principal directions, 5–5 mm proximally and distally (**b**)

A dedicated software package (QAngio® XA 3D Research Edition 1.0 program, Medis Specials bv, Leiden, The Netherlands) was used for 3D QCA and for 3D coronary artery reconstruction. Automated quantifications of the ACr and the bending angles of the proximal and distal edges of the target segment before and after stenting were performed both at end-diastole and end-systole. The appropriate frames were selected on the basis of the ECG traces. The arc was the midline of the analysed segment, while the chord was the distance between the proximal and distal edges of the analysed segment (Fig. 1a). To measure the bending angles at the edges of the target segment, we used two vectors along the centreline of the vessel towards the principal directions, 5–5 mm proximally and distally. The bending angle was defined as the angle of the two vectors subtracted from 180° (Fig. 1b). Both angles were measured during systole and diastole using images before (“pre-stent”) and after stenting (“post-stent”).

### Statistical analysis

All analyses were performed using the SPSS 20.0 for Windows (Statistical Product and Service Solutions, version 20, SPSS Inc., Chicago, IL, USA). Normality was assessed with normal probability (Q–Q) plot and with non-parametric Shapiro–Wilk test. All continuous variables were reported as the mean ± standard deviation, and Student *t*-test was used to compare groups. For values not following a normal distribution, the median and the interquartile range were expressed and compared between the groups using the Mann–Whitney *U* test. Univariate logistic regression analysis was performed to determine factors associating with ISR, and then multivariate logistic regression (forward stepwise, likelihood ratio test) was used to identify independent predictors of restenosis. Sex, diabetes mellitus, hypercholesterolaemia, smoking, nephropathy, hypertension and previous myocardial infarction were included in the analyses, together with the angiographic parameters of the proximal and distal pre- and post-stent bending angels, arc-chord ratios, as well as their changes. In the case of ACr, a convenient unit increase (1%) was used for the calculation of the odds ratio (OR) and its 95% confidence interval (CI). A linear regression model was used for analysing the relationship between each parameter. The cut-off value was determined with ROC analysis. As an index of statistical significance, a p value < 0.05 was accepted.

## Results

During the selected one year, 110 patients with repeat coronary angiography 3–30 months after stent implantation were screened. Sixty-four patients fulfilled all inclusion and exclusion criteria. Table 2 shows the clinical characteristics of the patients involved in the study. DES was implanted in 37 patients, while BMS was used in 27 patients. In our study cohort, 22 patients were diagnosed with ISR, while 42 patients without any restenosis served as control. The two groups did not differ regarding major cardiovascular risk factors, proportion of the treated vessel or the type of stent. However, age was higher in the ISR group.

**Table 2:** Clinical characteristics of the study patients

Parameters	In-stent restenosis (n = 22)	Control (n = 42)	p value
Age (years; mean ± SD)	69 ± 7	63 ± 10	**0.012**
Male (%)	46	57	0.37
Diabetes mellitus (%)	46	31	0.25
Hypercholesterolemia (%)	36	43	0.62
Smoking (%)	14	24	0.36
Nephropathy (%)	0	3	0.45
Treated hypertension (%)	76	89	0.19
Previous myocardial infarction (%)	14	24	0.36
Treated vessel (%)			0.186
LAD	37	57	
Cx	27	12	
RCA	36	31	
Stent type (%)			0.99
BMS	46	45	
DES	54	55	

As an index of statistical significance, a p value < 0.05 was accepted and marked bold

*LAD* left anterior descending, *Cx* left circumflex, *RCA* right coronary artery, *BMS* bare metal stent, *DES* drug-eluting stent

The assessments for the ACr and the bending angles were performed in both systole and diastole. There was no statistical difference in the average angles between the ISR and control groups. However, the pre-stent ACr was significantly higher in the ISR group (1.06 [IQR 1.03, 1.12] vs. 1.05 [IQR 1.03, 1.07], p = 0.04). Moreover, the change in ACr after stenting was also higher in the ISR group ( − 0.02 [IQR − 0.04, − 0.01] vs. − 0.01 [IQR − 0.03, 0], p = 0.03). These data are reported in Table 3.

**Table 3:** Results of 3D coronary analysis

Parameters	In-stent restenosis (n = 44)^a^	Control (n = 84)^a^	p value
Proximal pre-stent BA (°)	6.85 (IQR 4.83, 12.8)	6.85 (IQR 3.63, 9.9)	0.17
Proximal post-stent BA (°)	4.84 (IQR 2.43, 10.78)	5.05 (IQR 2.63, 8.48)	0.54
Change in proximal BA after stenting (°)	− 0.60 (IQR − 5.4, 3.78)	− 1.50 (IQR − 4.28, 1.75)	0.77
Distal pre-stent BA (°)	8.10 (IQR 2.93, 12.23)	6.50 (IQR 3.33, 10.9)	0.51
Distal post-stent BA (°)	5.05 (IQR 2.3, 8)	5.40 (IQR 2.73, 9.25)	0.64
Change in distal BA after stenting (°)	− 1.85 (IQR − 5.58, 1.55)	− 0.55 (IQR − 4.28, 2.85)	0.41
Pre-stent ACr (%)	106 (IQR 103, 112)	105 (IQR 103, 107)	**0.04**
Post-stent ACr (%)	104 (IQR 102, 108)	104 (IQR 102, 105)	0.36
Change in ACr after stenting (%)	− 2 (IQR − 4, − 1)	− 1 (IQR − 3, 0)	**0.03**

As an index of statistical significance, a p value < 0.05 was accepted and marked bold

*ACr* arc-chord ratio, *BA* bending angle, *IQR* interquartile range

^a^All cases were analysed twice, in systole and in diastole

**Fig. 2:**
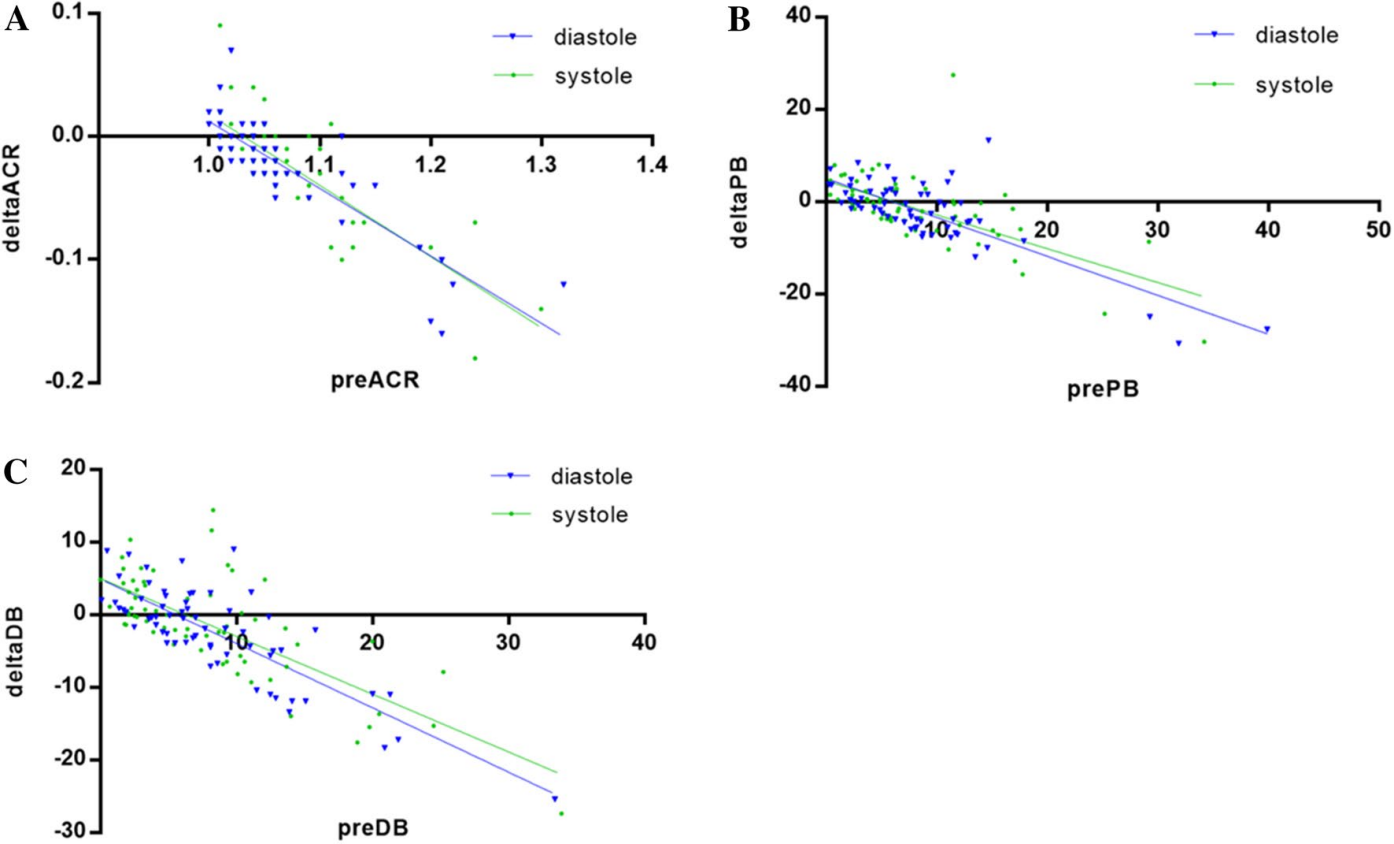
Correlation of pre-stent 3D values (**a***ACr* arc-chord ratio, **b***PB* proximal bending angle, **c***DB* distal bending angle) and their corresponding changes: X axis showing pre-sent values, Y axis showing the change in values after stent implantation (delta values = post-stent − pre-stent) in diastole (blue) and in systole (green)

**Fig. 3:**
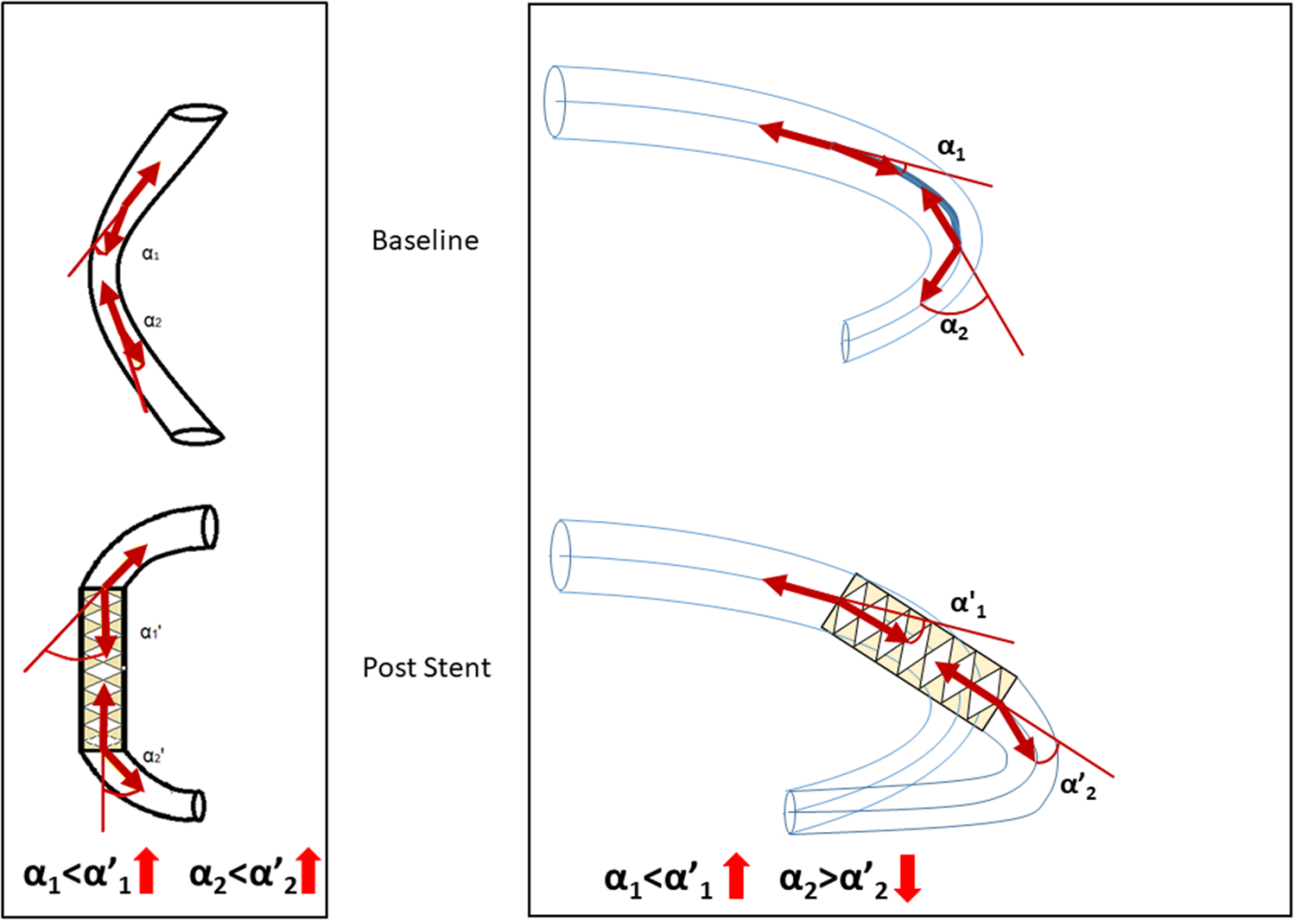
Comparison of a prior concept/left/ to our theory/right/ regarding the changes of bending angles at the edges of a stent (α_1_: proximal pre-stent angle, α_2:_ distal pre-stent angle, α′_1:_ proximal post-stent angle, α′_2:_ distal post-stent angle). Previously, the increase of edge angles was described as a consequence of an inflexible stent/left/. According to our concept, these changes are depending on the value of the pre-stent angle. Below 7° the edge bending angle usually increased (α_1_ < α’_1_), over 7° the angles were usually straightened after stent implantation (α_2_ > α’′_2_)/right/. The longitudinal straightening effect of the stent spread slightly over the edges of the stent in cases of decreased edge bending angles

Similar changes of the curvature have been observed with both stent types. The higher initial ACr values associated with more pronounced straightening of the curvature (DES: r =  − 0.83, p < 0.001; BMS: r =  − 0.86, p < 0.001) (Fig. 2a). Significant negative correlations were also shown for the proximal and distal edge bending angles (r =  − 0.7727, p < 0.00; r =  − 0.7190, p < 0.001, respectively) (Fig. 2b, c). Low ( < 7°) pre-stent edge bending angles often showed an increase after stent implantation, which can be explained by the newly generated buckling tendency at the edges of the stent in case of the straightening of the initially curved stented segment. On the other hand, the high initial values of the bending angle at the edges of the target segment associated with decreasing bending angles, presumably due to the slight overspreading of the stent’s longitudinal straightening effect beyond its edges (Fig. 3).

The univariate logistic regression analysis demonstrated that the pre-stent ACr and the percentile change in ACr after stenting correlated with the ISR (Table 4). However, the multivariate logistic regression modelling showed that only the pre-stent ACr was an independent predictor of ISR (odds ratio for 1% increase of the ACr: 1.08; p = 0.012).

**Table 4:** Univariate and multivariate modelling of parameters describing ISR

Variables	Univariate analysis OR (95% CI)	P value	Multivariate analysis (95% CI)	p value
Male	1.6 (0.77–3.33)	0.21		
Diabetes mellitus	1.86 (0.88–3.95)	0.11		
Hypercholesterolemia	0.76 (0.36–1.61)	0.48		
Smoking	0.52 (0.19–1.43)	0.2		
Nephropathy	0	0.999		
Treated hypertension	0.39 (0.14–1.08)	0.07		
Previous myocardial infarction	0.52 (0.19–1.43)	0.2		
Proximal pre-stent BA	1.03 (0.97–1.08)	0.33		
Distal pre-stent BA	1.03 (0.97–1.09)	0.32		
Pre-stent ACr	1.082 (1.017–1.15)	**0.012**	1.082 (1.017–1.15)	**0.012**
Proximal post-stent BA	1.06 (0.99–1.14)	0.11		
Distal post-stent BA	1 (0.92–1.08)	0.97		
Post-stent ACr	1.089 (0.982–1.207)	0.11		
Change in proximal BA after stenting	1 (0.96–1.06)	0.75		
Change in distal BA after stenting	0.98 (0.93–1.03)	0.35		
Change in ACr after stenting	0.89 (0.81–0.98)	**0.015**	0.948 (0.799–1.125)	0.54

As an index of statistical significance, a p value < 0.05 was accepted and marked bold

*ACr* arc-chord ratio, *BA* bending angle, *CI* confidence interval

The ROC analysis indicated a possible cut-off value at 1.055 for pre-stent ACr to predict ISR (AUC = 0.61; sensitivity = 59%, specificity = 60%).

**Fig. 4:**
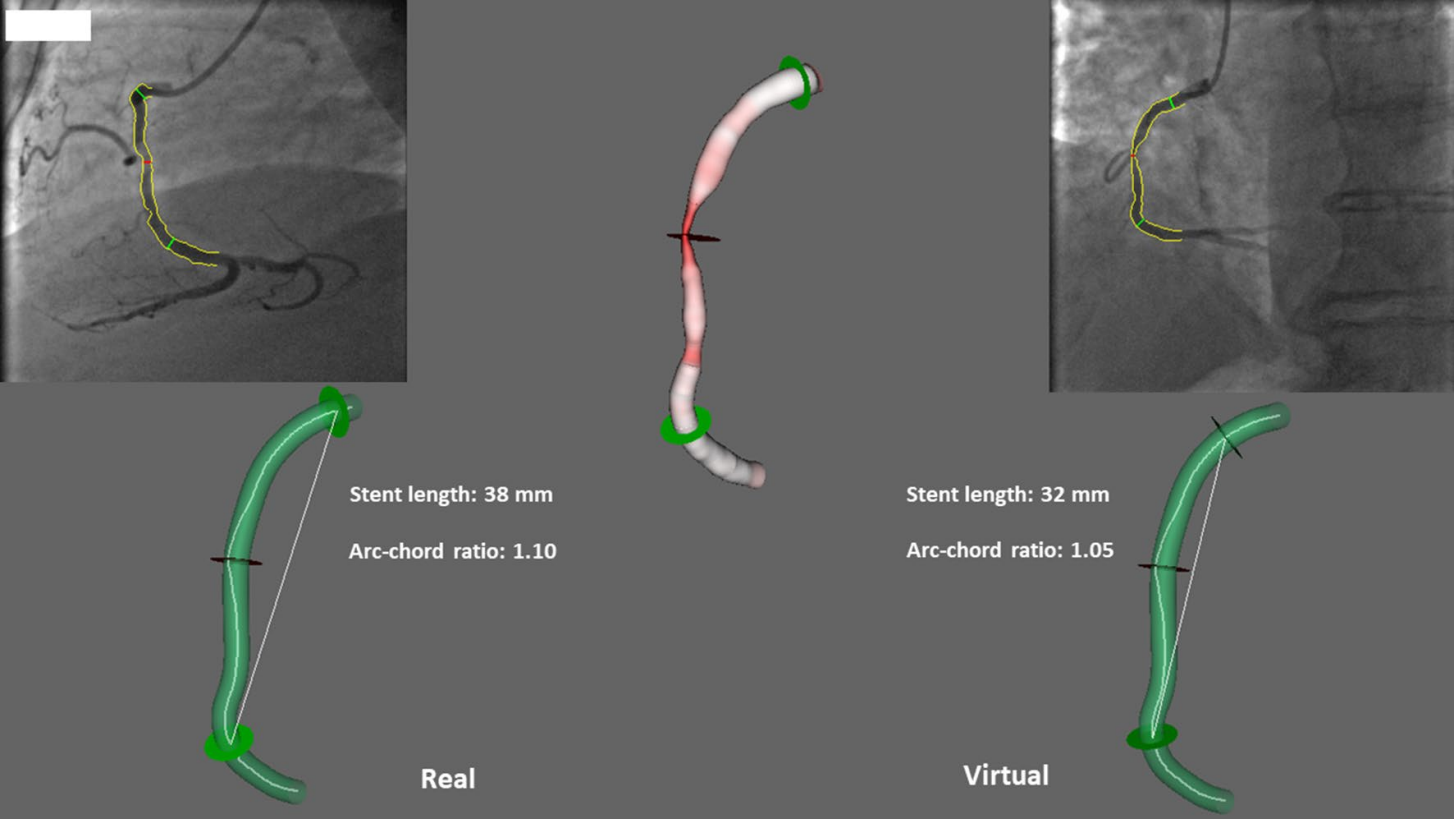
Virtual stenting: the original 1.10 pre-stent ACr in case of a 38 mm long stent can be decreased to 1.05 by choosing a 32 mm length

**Fig. 5:**
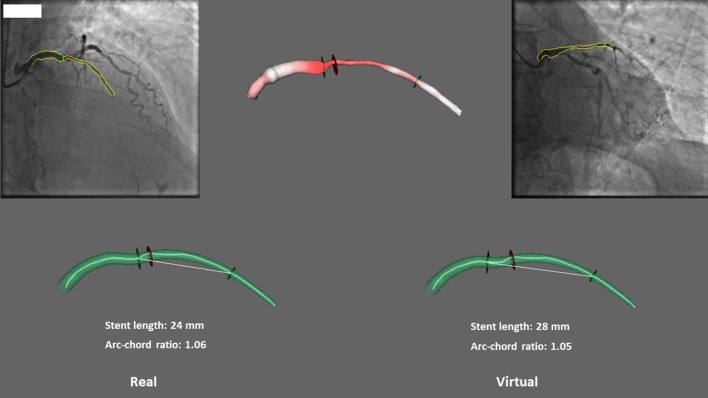
Virtual stenting: the 24 mm stent resulted in 1.06 ACr, while the 28 mm length would have generated a 1.05 ACr in a virtual position

By virtual stenting, it is possible to determine the ACr before the intervention for specific stent lengths (Figs. 4, 5). Generally, shorter length of the stent associates with lower ACr in a symmetrical bend (Fig. 4). However, in cases of curves with one straight (non-bending) arm, a rare situation can develop where a longer stent generates lower ACr. The possible beneficial effect of choosing a longer stent in the latter case can be explained by the less local stress induced by the stent to the vessel wall. This results in less intensity but more spreading of the stress along the curved vessel segment (Fig. 5).

## Discussion

At present, the exact mechanism of restenosis is not fully described. It is well known that stent implantation has an effect on the geometry of the coronary artery, but data regarding the impact of coronary angulation on restenosis is limited and controversial. Previous studies demonstrated that macroscopically, the stent placement induces straightening of the arterial segment [3, 11]. In animal model the rigid stent implantation increased the edge curvature of the stented segment by 121% and 100% [12]. In human investigation the straightening effect of the stent implantation was identified as a predictor of restenosis [3, 4]. However, Fukuda later concluded that the lesion angulation was not associated with restenosis following early generation sirolimus-eluted stent implantation [13]. Recently, Gomez-Lara et al. examined the role of bendings, vessel curvature and angulation in target vessel revascularisation with the comparison of two second-generation DES platforms. The authors demonstrated that the angulation of the lesion or the changes of the vessel angulation from pre to post-implantation did not correlate with target vessel revascularisation and target lesion failure at 1 year follow-up [14]. On the contrary, the restriction of the hinge motion after stent implantation was reported as a predictor of restenosis [15, 16].

It is important to notice that the above mentioned clinical studies used 2D analysis to determine vessel angulation and geometric changes. It is also known that overlapping and foreshortening are main limitations of the 2D QCA assessment [10]. These factors make an accurate spatial coronary reconstruction very difficult, which is essential in the determination of coronary curvature.

A recent investigation emphasised the advantages of 3D QCA by demonstrating that the stent implantation changes the natural tortuous course of coronary arteries, and the decrease in the coronary bending angle contributes to stent failure. The authors described coronary curvatures by measuring the maximum and mean bending angles at different time points. They showed that the mean systolic post-stent bending angle and the change in the mean systolic bending angle after stenting may be predictors of restenosis [17]. In that study the maximum and mean bending angles were chosen to describe the target segment’s bending angles. In our opinion it may be difficult to standardise the determination of the maximum or even the mean bending angle across a severely tortuous segment. Therefore we decided to use the ACr, which provides a more reliable characterisation of a tortuous coronary segment, even if it contains multiple curvatures [18].

At the moment, it is unclear how the geometric change following stent implantation provokes restenosis development, and what the exact pathomechanism of restenosis could be. Given that the endothelium is able to produce anti-atherogenic substances (e.g. nitrogen monoxide and endothelin) in response to shear stress, it is generally accepted that one of the main contributors of stenosis development in native coronary arteries is the presence of a pathologically low shear stress [19–23]. Also, several precise computational fluid dynamics models described that vessel bending changes have significant effect on local haemodynamics resulting in altered endothelial shear stress [24]. However, the role of shear stress in stenosis development after stent implantation is more ambiguous. Based on available data, a recent review rose the question whether intimal hyperplasia is part of the healing process after stent implantation or a predictor of later clinical restenosis [9]. It is reasonable to analyze not only the flow parameters but also the stress and strain in the vessel wall generated by the radial stretching effect of the implanted stent [25]. As for the magnitude of these forces, the normal value of the average wall shear stress in a coronary artery is considered approximately 1 Pa only, while the stretching effect of an implanted stent could generate even 3 × 10^5^ times higher circumferential stress [26].

In line with previous literature data, we demonstrated that the curvature of the target coronary artery segment significantly decreased after stenting due to the straightening effect of the stent. In contrast to some previous studies [8, 10, 27], our results did not support the idea that the straightening effect of the stent always generates an increase of the angles at the edges of the stent. In particular, we observed an increase after stenting in cases of pre-stent edge bending angles < 7°. In these cases, the straightening of the stented coronary artery segment generated a buckling tendency at the edges of the stent, which was in line with previous observations. On the other hand, bending angels generally decreased in cases of higher initial values due to the longitudinal straightening effect spreading slightly over the edges of the stent.

In our study, the predictor of restenosis was the pre-stent ACr. If the pre-stent ACr was higher than 1.055, the risk of ISR increased. While the relatively low sensitivity and specificity of this cut-off value reflect the multifactorial nature of restenosis, our results may propose a new concept for stent positioning in a curved coronary artery segment.

In cases of high ACr, the development of restenosis could be explained by the increased wall stress. According to Hook’s law, the force due to the bending of the stent will be proportionate to the initial ACr. In our hypothesis, the force due to the bending of the stent is one of the main source of the increased wall stress after stenting, which is as an important facilitator of abnormal intimal proliferation [28, 29].

Therefore, the clinical importance of appropriate stent length regarding ACr should be considered. Our two examples from the restenotic group stented virtually demonstrate possibilities for decreasing the ACr by shortening or extending the chosen length of the stent (Figs. 4, 5).

## Limitations

Our retrospective observational study was performed on a relatively small sample size. The statistical power was achieved by pooling cases with DES and BM stents implantations, despite the fact that DES have less re-stenosis rate. However, the stent platform was restricted to that of cobalt-chromium, which assured a quite homogeneous group regarding the mechanical characteristics of the stents.

For the quantification of the coronary angiography, generally the diastolic frame is suggested. In our study, the systolic and the diastolic frames were analysed separately, but the angiographic parameters from the two heart cycles were pooled for further analysis. This approach may be justified by the fact that the bending of the vessel curve can change differently in various localisations, therefore the separate systolic and diastolic analyses are reasonable, while taking into account the localisation of the investigated coronary segment [30]. Unfortunately, in our small sized patient population this approach would not be feasible for statistical reasons.

## Conclusions

Following stent implantation, the changes of the angles at the ends of the stent correlate with the initial local bending angles, but not with the decrease in the stented segment’s bend. The ACr characterises the spatial curve of the target coronary artery segment well, while its value determines the extent of chronic stress in the vessel wall, possibly leading to pathological intimal proliferation. In specific cases, the initial ACr can be modified towards a more favourable value by appropriate choice of stent length and position.
